# The iron load of lipocalin-2 (LCN-2) defines its pro-tumour function in clear-cell renal cell carcinoma

**DOI:** 10.1038/s41416-019-0655-7

**Published:** 2019-11-27

**Authors:** Claudia Rehwald, Matthias Schnetz, Anja Urbschat, Christina Mertens, Julia K. Meier, Rebekka Bauer, Patrick Baer, Sofia Winslow, Frederik C. Roos, Klaus Zwicker, Arnaud Huard, Andreas Weigert, Bernhard Brüne, Michaela Jung

**Affiliations:** 1 Institute of Biochemistry I, Goethe-University Frankfurt, Theodor-Stern-Kai 7, 60590 Frankfurt am Main, Germany; 2 Institute for Biomedicine, Aarhus University, C. F. Møllers Allé 6, 8000 Aarhus, Denmark; 3 Clinic of Urology and Pediatric Urology, Philipps-University Marburg, Baldingerstraße, 35043 Marburg, Germany; 4 Division of Nephrology, Department of Internal Medicine III, Goethe-University Frankfurt, Theodor-Stern-Kai 7, 60590 Frankfurt am Main, Germany; 5 Clinic of Urology, Goethe-University Frankfurt, Theodor-Stern-Kai 7, 60590 Frankfurt, Germany; 6 German Cancer Consortium (DKTK), partner site Frankfurt/Mainz, Germany; 7 Frankfurt Cancer Institute, Goethe-University Frankfurt, 60596 Frankfurt, Germany; 8 Project Group Translational Medicine and Pharmacology TMP, Fraunhofer Institute for Molecular Biology and Applied Ecology, 60596 Frankfurt, Germany

**Keywords:** Cancer metabolism, Renal cell carcinoma

## Abstract

**Background:**

We aimed at clarifying the role of lipocalin-2 (LCN-2) in clear-cell renal cell carcinoma (ccRCC). Since LCN-2 was recently identified as a novel iron transporter, we explored its iron load as a decisive factor in conferring its biological function.

**Methods:**

LCN-2 expression was analysed at the mRNA and protein level by using immunohistochemistry, RNAscope® and qRT-PCR in patients diagnosed with clear-cell renal cell carcinoma compared with adjacent healthy tissue. We measured LCN-2-bound iron by atomic absorption spectrometry from patient-derived samples and applied functional assays by using ccRCC cell lines, primary cells, and 3D tumour spheroids to verify the role of the LCN-2 iron load in tumour progression.

**Results:**

LCN-2 was associated with poor patient survival and *LCN-2* mRNA clustered in high- and low-expressing ccRCC patients. LCN-2 protein was found overexpressed in tumour compared with adjacent healthy tissue, whereby LCN-2 was iron loaded. In vitro, the iron load determines the biological function of LCN-2. Iron-loaded LCN-2 showed pro-tumour functions, whereas iron-free LCN-2 produced adverse effects.

**Conclusions:**

We provide new insights into the pro-tumour function of LCN-2. LCN-2 donates iron to cells to promote migration and matrix adhesion. Since the iron load of LCN-2 determines its pro-tumour characteristics, targeting either its iron load or its receptor interaction might represent new therapeutic options.

## Background

Renal cell carcinoma (RCC) accounts for 3% of all cancers, 90% of all kidney cancers and is the third most recurrent urologic tumour. Due to the complex anatomy and histology of the kidney, its neoplastic transformation causes a plethora of RCC subtypes that differ in cellular origin, histology and prognosis.^1^ The three main RCCs are clear-cell RCC (ccRCC), papillary RCC (pRCC) and chromophobe RCC (chRCC). The majority of RCC is detected incidentally, with curative partial or total nephrectomy representing the first therapeutic approach for RCC.^1^ Therapeutic options are currently limited for metastatic disease, since RCC is chemo- and radiation resistant. The first-line treatment in patients with metastatic disease includes systemic therapy by using multitarget tyrosine kinase inhibitors (TKIs) and mammalian target of rapamycin (mTOR) inhibitors. Despite their promising clinical activity, disease outcome still remains poor and future therapeutic directions point to the advantage of combinatory immunotherapy with checkpoint inhibitors, such as PD-1 monoclonal antibody.^2^ However, there is still an urgent need to identify new targets and players in RCC pathogenesis and progression.

Since lipocalin-2 (LCN-2) represents an already-established biomarker for acute and chronic kidney pathologies, we aimed at elucidating its role in renal cancer. LCN-2 is a 25-kDa protein of the lipocalin superfamily that is rapidly upregulated after renal tubular injury.^3,4^ Application of exogenous LCN-2 to renal epithelial cells induced proliferation and expression of genetic markers of early epithelial progenitors, suggesting that LCN-2 may act as a growth and differentiation marker.^5^ Moreover, we recently found that macrophage-derived LCN-2 mediates cytoprotective mechanisms in an ischaemia/reperfusion model, thereby promoting renal regeneration.^6,7^ However, LCN-2 is also expressed in tumour-associated macrophages (TAM) upon contact with apoptotic tumour cells.^8^ Macrophage-derived LCN-2 promoted tumour progression and metastasis in experimental mammary tumours.^9,10^ In line, overexpression of LCN-2 is associated with reduced disease-free survival in breast cancer patients^11^ and enhanced tumour progression, whereas depletion of LCN-2 inhibited tumour growth, neovascularisation, and metastasis in experimental mammary tumour models.^8,10,12,13^ Mechanistically, the production of LCN-2 was linked to apoptotic cell-secreted sphingosine-1-phosphate (S1P) and the downstream activation of the STAT3 pathway.^8^ Yet, mechanistic details regarding the function of LCN-2 in RCC development are still lacking. So far, studies focused on the evaluation of serum and urinary LCN-2 as a biomarker for disease progression.^14,15^ Interestingly, it was speculated that the functional outcome of LCN-2 largely depends on its iron load. Previous studies pointed out that LCN-2 ameliorates ischaemia/reperfusion injury by supplying proximal tubule cells with iron.^16,17^ Along the same lines, it was described that exclusively iron-loaded LCN-2 triggers cell survival upon internalisation.^18^ Interestingly, we described LCN-2 as a major iron transporter, which, in turn, enhanced tumour cell proliferation.^19^ Although its ability to bind and transport iron seems to be the crucial mechanism of how LCN-2 promotes cell survival, inhibits the inflammatory outcome and reduces tubular apoptosis during acute renal injuries, the decisive role of the iron load of LCN-2 has not been investigated so far in renal cancer.

In this study, we provide evidence that the iron load of LCN-2 might play a decisive role for the pro-tumour characteristics in ccRCC. These findings offer the possibility of novel treatment strategies that interfere with and/or block the iron-binding ability of LCN-2, whereby tumour growth might be significantly reduced.

## Methods

### Cell culture

Human renal cancer cell lines CAKI 1, 786-O, A498, and RCC4 cells were a kind gift of PD Dr. Anja Urbschat and were cultured in Dulbecco’s modified Eagle’s medium (DMEM; Gibco, Dreieich, Germany, 41965) with high glucose, supplemented with penicillin (100 U/ml) (Sigma Aldrich, Taufkirchen, Germany, P4333), streptomycin (100 mg/ml) (Sigma Aldrich, S8636) and 10% FCS (Capricorn Scientific, Ebsdorfergrund, Germany, FBS-11A). RCC4 cell medium was further supplemented with 1 mM sodium pyruvate and 1× MEM nonessential amino acids (Gibco, 11140). Routinely, cells were tested with VenorGeM Classic (Minerva Biolabs, Berlin, Germany, 11-1100) for mycoplasma contamination. Cells were kept in a humidified atmosphere with 5% CO_2_ at 37 °C, and cell lines were passaged every 2–4 days. For experiments, passages from 4 to 28 were used.

### Spheroid experiments

Renal carcinoma cell CAKI1 spheroids were generated in 1.5% agarose-coated 96-well plates (Greiner Bio-One, Frickenhausen). In total, 5000 cells were used per well. Spheroids were stimulated with holo-, apo- or a non-iron-binding LCN-2 mutant (1 µg/ml) every second day for 12 days. Images were taken on the days of stimulation, and the spheroid diameter was measured by using ImageJ software. Spheroids were carefully picked and subsequently either processed for RNA isolation as described below or used for invasion assays.

For the invasion assay, three spheroids of each stimulation group were embedded in a collagen I matrix for 4 days. Images were taken every day of each spheroid from each group from at least four independent experiments. Representative pictures are given.

### Tumour tubular epithelial cell isolation

Human renal tumour tubular epithelial cells (T-TEC) were isolated as previously described.^20,21^ Briefly, tumour tissue was minced and digested with collagenase/dispase (1 mg/ml) for 45 min at 37 °C. Digested fragments were passed through a 106 -µm mesh and incubated with collagenase (1 mg/ml), DNase (0.1 mg/ml) and MgCl_2_ (5 mmol/l) for 45 min at 37 °C. The cell pellet was washed with Hank’s buffered salt solution after Percoll density-gradient centrifugation. Cells were seeded on FCS-precoated plates and grown in M199 medium (Sigma Aldrich, M4530) supplemented with penicillin (100 U/ml), streptomycin (100 mg/ml) and 10% FCS. For the first 2–3 days after isolation, the antibiotic meropenem (100 µg/ml, Sigma Aldrich, M2574) was added to the culture medium. T-TEC medium was changed every 3 days, and cells were passaged when confluence was reached. Passages between 2 and 4 were used for experiments.

### Participants

Both study protocol and consent documents were approved by the local Ethic Committee of the Goethe-University Hospital Frankfurt am Main (file number 04/09 UGO 03/10) and Philipps-University Hospital Marburg (file number 122/14). Prior to surgery, patients gave their written informed consent. Tumour and the corresponding adjacent healthy tissues, if possible, were obtained from 41 patients with histopathological diagnosis of renal cancer (see Table 1). Preoperative staging was accomplished for all patients either by imaging by CT and/or MRI and surgery was performed before receiving other therapies. Tumour and adjacent healthy renal tissue were collected immediately after nephrectomy between 2016 and 2019. No follow-up data were available so far. Samples were aliquoted and immediately processed for single-cell suspensions, fixed in 4% paraformaldehyde (PFA) for immunohistological analysis or stored at −80 °C until further processing. The current UICC TNM classification of malignant tumours was applied for pathological examination.

**Table 1:** Patient cohort.

Parameter	
Number of patients	32
*Age (years)*	
Mean	65 ± 10
Median	66 ± 10
Range	44–84
*Sex*	
Female	22%
Male	78%
*pT stage*	
pT1–pT2	56%
pT3–pT4	44%
*Grade*	
G1–G2	84%
G3–G4	16%
*Nephrectomy*	
Radical	66%
Partial	34%

Patient cohort is composed of 41 patients, grouped into three major renal tumour types: ccRCC, pRCC and chRCC. Patient parameters such as age, sex, pT stage, grade and surgery type are depicted in the table

### RNA extraction and quantitative real-time PCR (qPCR)

RNA isolation, cDNA synthesis and qPCR were performed as previously described.^19^ Briefly, RNA was isolated (peqlab, Darmstadt, Germany, 30-1010) and transcribed into cDNA (Thermo Fisher, Dreieich, Germany, K1642), serving as a template in a qPCR mix (Bio-Rad, Munich, Germany, 1725006CUST). 18S mRNA expression served as an internal housekeeping gene control. Primers were bought from Bio-Rad (*LCN-2*; qHsaCED0045408) or from Biomers (Ulm, Germany, *18* *S rRNA* 5′→3′ GTA-ACC-CGT-TGA-ACC-CCA, 3′→5′ CCA-TCC-AAT-CGG-TAG-TAG-CG; *LCN-2R* 5′→3′ GCT-CTT-CGT-GGC-TCT-GGG-CAT, 3′→5′ TGG-CAT-TGG-GAG-GCT-GCT; *fibronectin* 5′→3′ GTG-ACC-GAA-ATC-ACA-GCC-AGT-AG, 3′→5′ TAC-TGT-GGC-TCA-TCT-CCC-TCC-TCA; *Snail* 5′→3′ CGC-CTC-CAA-AAA-GCC-AAA-CTA, 3′→5′ GCT-GAG-GAT-CTC-TGG-TTG-TGG-TAT; *CDH1* 5′→3′ TTC-CTC-CCA-ATA-CAT-CTC-CC, 3′→5′ TTG-ATT-TTG-TAG-TCA-CCC-ACC; *CD24* 5′→3′ CGC-GCC-CAG-CCA-TCA-AAA-T, 3′→5′ GCT-TCC-AGT-CTT-CAC-TTC-CCA-AAT-AC; *CD44* 5′→3′ AGC-AAC-TGA-GAC-AGC-AAC-CAA-GAG-G, 3′→5′ CCA-GCC-ATT-TGT-GTT-GTT-GTG-TGA-A.

### LCN-2 immunoprecipitation (IP)

Immunoprecipitation was performed according to the protocol previously described by Mertens et al.^19^ Human tissue samples were snap frozen, homogenised and sonicated. Unlysed material was removed by centrifugation, and the supernatant was subjected to LCN-2 IP by applying an LCN-2 specific antibody (R&D systems, Wiesbaden, Germany, MAB1757) and bound to magnetic beads (Thermo Fisher, 10004D). After elution from the beads, iron content of samples was analysed by atomic absorption spectrometry.

Equal amounts of 50 µl from whole-cell lysate (WCL), IP eluate, IP wash step, and eluted IgG-treated beads were denatured and subjected to gel electrophoresis. Protein transfer to a PVDF membrane was analysed by Ponceau-S stain (Sigma Aldrich, P3504), and specific detection of LCN-2 by western blot analysis was performed by using an LCN-2 antibody (R&D systems, MAB1757; 1:500 in 2.5% BSA in TBS-T). The membrane was incubated with IRDye secondary antibody (LI-COR, Bad Homburg, Germany, 926-32219; 1:5000 in 5% BSA/TBS-T) for subsequent protein detection and visualisation by Odyssey infrared imaging system.

### Atomic absorption spectrometry (AAS)

Iron measurements were performed as previously described.^19^ Patient-derived whole-tissue homogenates were either measured as whole homogenates or underwent LCN-2 IP with the final eluate being analysed for its iron content, which was normalised to the total whole homogenate/IP protein amount. In order to define the amount of LCN-2-bound iron within the pool of total iron in tumours, we calculated the amount of LCN-2-bound iron as well as the total amount of iron in whole homogenates per 1 × 10^6^ cells and determined the fraction of LCN-2-bound iron in the total iron pool of the respective means.

For cell culture experiments, cells were harvested in lysis buffer (62.5 mM Tris-HCl, pH 6.8, 50% glycerol and 2% SDS). The iron amount was quantified relative to the total protein content and normalised to untreated control.

### Perl’s stain

Perl's stain was performed according to the manufacturer’s kit protocol (Sigma Aldrich, HT20). Briefly, 4 -µm tissue sections were deparaffinised and hydrated before staining in ‘Working Iron Stain Solution’ for 30 min. After rinsing in deionised water, slides were incubated for 2 min in nuclear fast red solution. After washing for 5 min under running water, slides were rapidly dehydrated in 95 and 100% EtOH, before incubating for 15 min in xylol and mounted. Pictures were acquired on an Axioskop 40 (Zeiss, Oberkochen, Germany).

### Database analysis

For violin blot visualisation of *LCN-2* expression, gene expression data of the Cancer Genome Atlas were selected (https://portal.gdc.cancer.gov/) and analysed.

For Kaplan–Meier overall survival curves for patients with high and low expression of *LCN-2* and *LCN-2R*, the R2 Genomics Analysis and Visualization Platform^22^ (http://r2.amc.nl) was employed. The ‘Tumor Kidney Renal Clear Cell Carcinoma - TCGA – 533’ data set was used for visualisation by using the Kaplan Scan tool of the R2 Genomics Analysis and Visualization Platform, where the optimum survival cut-off is established based on statistical testing within the web application. The scanner separates samples of the selected data set based on gene expression cut-offs into two groups and tests the *p*-value in a log-rank test. Default settings of the Kaplan Scan including a log-rank comparison between the groups were used to determine an optimum survival cut-off as described in the portal. The resulting *p*-value as well as the Bonferroni correction of the log-rank comparison are included in the plots. The numbers in parentheses indicate the number of individuals in each group.

### Vector generation of non-iron-binding LCN-2 mutant

QuikChange II XL Site-Directed Mutagenesis Kit (Agilent Technologies, Waldbronn, Germany, 200522) was used to subsequently insert point mutations into the pGEX-4T3_LCN-2 plasmid, resulting in an amino acid exchange. Following PCR, the parental DNA template was digested by using *Dpn I* (New England Biolabs, Frankfurt (Main), Germany, R0176). The mutated plasmids were transformed into XL10 Gold ultracompetent cells (Agilent Technologies, 200314). Single clones were used for plasmid amplification and isolation, by applying the NucleoSpin Plasmid kit (Macherey&Nagel, Düren, Germany, 740588). Successful point mutations were confirmed by sequencing. Arginine 81 was mutated to glycine 81, lysine 125 to glutamine 125 and lysine 134 to glutamine 134, finally yielding the pGEX-4T3_LCN-2_mutant plasmid.

### Generation of recombinant LCN-2 and complex formation

Recombinant human LCN-2 was produced by transformation of *E. coli* (New England Biolabs, C2527H) with either a pGEX-4T3-LCN-2 or the pGEX-4T3_LCN-2_mutant plasmid, both containing an insert for human *LCN-2* tagged with glutathione-S-transferase (GST) for purification. LCN-2 expression of bacterial culture was initiated by supplementing Isopropyl-β-D-thiogalactopyranosid (IPTG, Sigma Aldrich, I6758). LCN-2 was purified by using Pierce Glutathione Agarose (Thermo Fisher, 16100). Detoxi-Gel Endotoxin Removing Resin (Thermo Fisher, 20339) was utilised for eliminating bacterial endotoxins.

LCN-2 iron–catechol complex formation was monitored by UV–Vis spectrometry in the range of 300–800 nm. The iron-free (apo-) protein spectrum was obtained from a 6.25 µM LCN-2 solution. For acquisition of spectra of both iron-loaded (holo-) LCN-2 and mutant LCN-2, the protein solution was mixed with an aliquot of 5 mM iron–catechol (3:1 mol:mol) solution, resulting in equimolar concentration of LCN-2 and iron–catechol.

### Adhesion assay

Cells were stimulated with 1 µg/ml recombinant human LCN-2 for 24 h and marked with cell tracker green (Invitrogen, Dreieich, Germany, C7025). Afterwards, cells were counted and seeded onto collagen I (10 µg/ml; Thermo Fisher, CB354249) or fibronectin (10 µg/ml; Sigma Aldrich, F1141) precoated wells for 2 h, washed and fixed with 4% PFA. Five pictures were taken from each group from at least three independent experiments by using triplicates. The number of attached cells was determined by using ImageJ automated makro-based analysis (National Institutes of Health, Bethesda, USA).

### Migration assay

The RTCA DP xCELLigence instrument (OLS, Bremen, Germany) was used to measure the migration of cells. Cells were added into the upper chamber of a two-chamber CIM plate (OLS) and stimulated with apo-, holo- or mutant LCN-2 (1 µg/ml). Continuous recording of migration was accomplished for 24 h. Data are displayed as a measure for time-dependent impedance changes, which is represented as the normalised slope per hour (slope 1/h) of the normalised cell index. The RTCA Software 1.2 (OLS) was used for acquisition and analysis.

### Proliferation assay

Proliferation of human CAKI 1 and primary patient-derived T-TEC cancer cells was measured by using the RTCA DP xCELLigence instrument (OLS) as described previously.^8^ Cells were stimulated with apo-, holo- or mutant LCN-2 (1 µg/ml). Continuous recording of proliferation was accomplished for 72 h, and data are displayed as time-dependent impedance changes, represented as the normalised slope per hour (slope 1/h) of the normalised cell index. The RTCA Software 1.2 (OLS) was used for acquisition and analysis.

### Immunocytochemistry

Cells were stimulated with recombinant human LCN-2 (1 µg/ml) for 3 h, washed, fixed with 4% PFA and permeabilised with 0.1% Triton X 100 (CARL ROTH, Karlsruhe, Germany, 3051). After blocking with 3% BSA (CARL ROTH, 8076) in PBS, primary antibody against the GST-tag (Thermo Fisher, 13-6700; 1:250 in 1% BSA/PBS) of LCN-2 was applied overnight at 4 °C. AF488-coupled anti-mouse secondary antibody (Life Technologies, Dreieich, Germany, A11001; 1:1000 in 1% BSA/PBS) was incubated for 1 h at room temperature. Before mounting (Southern Biotech, Eching, Germany, 0100-01), nuclei were stained with DAPI (Sigma Aldrich, D9542). Images were acquired on an LSM 800 (Zeiss) confocal microscope and evaluated by using ImageJ software.

### RNAscope

In situ hybridisation by RNAscope® technique was performed according to the manufacturer’s instructions (Advanced Cell Diagnostics (ACD), Newark, CA, USA). Four-micrometer paraffin-embedded patient tissue sections were deparaffinised and treated with H_2_O_2_, followed by antigen retrieval and protease treatment according to the RNAscope® Multiplex Fluorescent v2 assay kit’s instructions. For human kidney (normal and tumour) sections, probes to Hs-LCN-2 (ACD, 559441) and negative controls (3-plex negative control probe (DapB), ACD, 320871) were used. Probes were hybridised for 2 h followed by three amplification steps. The signal was detected with RNAscope® Multiplex fluorescent detection reagent kit v2 (ACD, 323110) by using Opal dyes (PerkinElmer, Rodgau, Germany).

### Immunohistochemistry

Immunohistochemistry was adapted from previously described protocols^8,19^ with antibodies recognising LCN-2 (R&D Systems, MAB1757; 1:100) with the Opal 4-color Automation IHC Kit (PerkinElmer, NEL820001KT) on 4-µm paraffin-embedded patient tissue sections. As secondary antibody for the LCN-2 antibody, a HRP-coupled anti-rat antibody (GE Healthcare, Solingen, Germany, NA935V; 1:500) was employed. Vectra automated imaging system (PerkinElmer) was used for image acquisition, while analysis was accomplished by using inForm software (PerkinElmer). First, nuclei were defined by DAPI fluorescence, whereby the total number of cells was obtained. Afterwards, cell size around the nucleus was adjusted, by defining the area of quantification, and a threshold for positive staining was determined. The software then counted the overall number of cells by DAPI fluorescence and the number of positive cells, resulting in a percent ratio of positive cells.

### Flow cytometric analyses

Patient tissue was dissociated by using the human Tumor Dissociation Kit (Miltenyi Biotech, Bergisch Gladbach, Germany, 130-095-929) and the GentleMACS system (Miltenyi Biotech). Sample acquisition was done on a LSRII/Fortessa flow cytometer (BD, San Jose, CA, USA) expressed as mean fluorescence intensity (MFI). Both antibodies and secondary reagents were titrated to determine optimal concentrations. CompBeads (BD) were used for single-colour compensation to create multi-colour compensation matrices. For gating, fluorescence minus one (FMO) controls were used. Cytometer Setup and Tracking beads (BD) were used for daily control of instrument calibration. Staining of patient-derived single-cell suspensions contained CD45 AF700 (Biolegend, San Diegeo, CA, USA, 368513) to exclude immune cells and CD326 PE-CF594 (BD Biosciences, San Jose, CA, USA, 565399) to gate CD326^+^ healthy and tumour epithelial cells, respectively. LCN-2R (Thermo Fisher, PA5-20543) was stained intracellularly in combination with an AF546-labelled secondary antibody (Life technologies, A-11035), after fixation and premeabilisation with Cytofix/Cytoperm (BD, 554714).

### Statistical analysis

Statistical analysis was performed by using Prism software (GraphPad Inc., San Diego, CA, USA). The Kolmogorov–Smirnov test was used to check for normal distribution. Accordingly, non-Gaussian distributed samples were analysed by applying the Wilcoxon signed-rank test. Data sets with normal distribution were analysed by using two-tailed paired Student’s *t* test, and were not corrected for multiple testing. Cell culture experiments were performed at least five times (independent experiments using three technical replicates). Graphs depict the means ± SEM. *P-*values were considered significant at *p < 0.05, **p < 0.01, ***p < 0.001.

## Results

### LCN-2 mRNA expression is unaltered in ccRCC

**Fig. 1:**
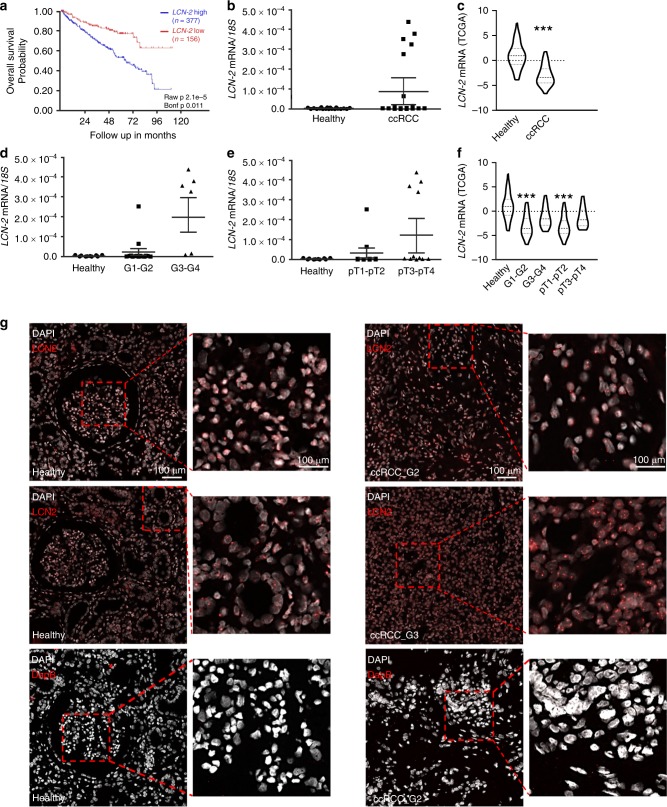
LCN-2 mRNA expression in human clear-cell renal cell carcinoma samples. **a** Kaplan–Meier curve of high or low *LCN-2* expression, number of patients in brackets. R2 bioinformatics platform^22^ was used to probe available TCGA data on ccRCC (KIRC data set). **b** *LCN-2* mRNA expression normalised to the housekeeping gene *18* *S* of whole-tissue homogenate of matched renal tumour and adjacent healthy tissue (*n* ≥20). **c** Analysis of *LCN-2* mRNA expression by using the TCGA database, applying the KIRC data set. **d** Expression of *LCN-2* mRNA of higher differentiated tumours (G1–G2) and less differentiated tumours (G3–G4) in comparison with healthy tissue. **e** *LCN-2* mRNA expression of healthy tissue compared with lower (pT1–pT2) and higher (pT3–pT4) T stage (*n* ≥20). **f** Association of tumour stage and *LCN-2* mRNA expression analysed in TCGA KIRC data set. **g** Representative pictures of ccRCC tumour and healthy tissue showing *LCN-2* transcripts (red) with RNAscope^®^ in situ hybridisation and DAPI (white) as nuclear stain. Representative pictures from two ccRCC patients (one G2 and one G3) with their respective healthy controls are shown. Staining for DapB served as control staining. Red rectangle defines the area for the enlarged detailed picture (*n* = 4). Graphs are displayed as means ± SEM with *p < 0.05.

In order to verify the role of LCN-2 in renal cancer, we first analysed the survival probability of ccRCC patients by applying the R2 Genomics Analysis and Visualization Platform using the cancer genome atlas (TCGA) data, including 533 patients of the KIRC data set. We found a significantly reduced patient survival probability associated with higher *LCN-2* expression (Fig. 1a). Therefore, we measured *LCN-2* mRNA expression in our own patient cohort (Table 1). We did not observe significant changes by comparing tumour tissue with adjacent healthy control tissue (Fig. 1b). However, we determined two populations of patients, one having a very high *LCN-2* mRNA expression and one with a very low expression. These observations could not be confirmed upon analysing the TCGA KIRC cohort, where a significant decrease in *LCN-2* mRNA expression was determined in ccRCC patients compared with healthy controls (Fig. 1c). By analysing the association of *LCN-2* mRNA expression to tumour grade (Fig. 1d) and tumour stage (Fig. 1e) in our small cohort, we found that LCN-2^high^ patients were associated with enhanced tumour grades and stages. By analysing *LCN-2* expression related to tumour grade and tumour stage using the KIRC data set in the TCGA database, we observed only small change towards higher LCN-2 expression in high-grade/high-stage tumours (Fig. 1f). We then performed RNAscope® in situ hybridisation to detect *LCN-2* transcripts in ccRCC tissues compared with adjacent healthy tissue of our own cohort and to localise it within the tissue (Fig. 1g). In tumour tissue, we observed equal and in most of the cases a higher number of overall *LCN-2* reactivity, with the notion that staining of *LCN-2* mRNA was even enhanced in high-grade tumours. Specificity of the RNA probe was tested by staining for DapB (negative control).

### LCN-2 protein expression is elevated in ccRCC and determines patient outcome

**Fig. 2:**
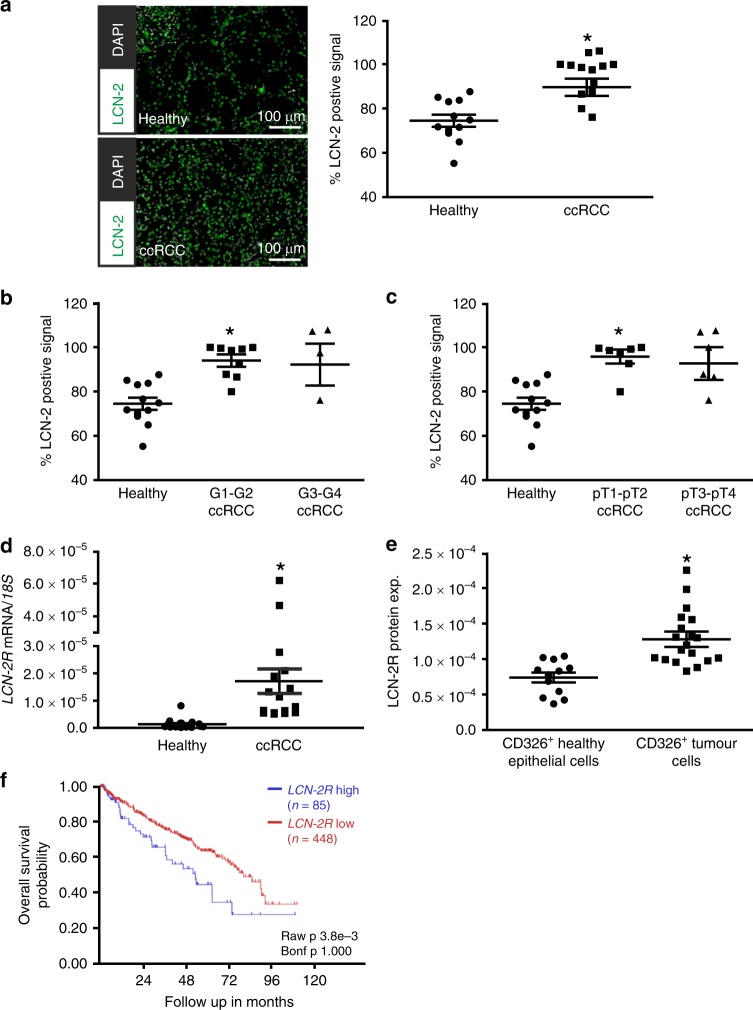
LCN-2 protein is elevated in ccRCC. **a** Healthy and tumour tissue were stained for LCN-2 (green). Nuclear counterstain with DAPI is displayed in white. Representative pictures are given (left), and the quantification of % LCN-2-positive signal was calculated (right). Six pictures of *n* ≥11 patients were quantified, respectively. **b** Correlation of the LCN-2-positive signal in higher differentiated tumours (G1–G2) and less differentiated tumours (G3–G4). **c** Percentage of positive cells to lower (pT1–pT2) and higher (pT3–pT4) T stage in comparison with healthy tissue (*n* ≥11). **d** *LCN-2R* mRNA expression normalised to the housekeeping gene *18* *S* of whole-tissue homogenate of matched renal tumour and adjacent healthy tissue. **e** Living single cells of healthy and tumour tissue were analysed by FACS. CD45^+^ immune cells were separated from CD326^+^ epithelial/tumour cells, which were subsequently analysed for LCN-2R expression, displayed as MFI (mean fluorescence intensity). **f** Kaplan–Meier curve of high or low *LCN-2R* expression, the number of patients in brackets. R2 bioinformatics platform^22^ was used to probe available TCGA data on ccRCC (KIRC data set). Graphs are displayed as means ± SEM with *p < 0.05.

We then determined LCN-2 protein expression in ccRCC tissues compared with adjacent healthy control tissue. An overall significantly elevated positive signal for LCN-2 protein was observed in tumour tissue compared with healthy control tissue by using immunofluorescence staining (Fig. 2a, left). Quantification of the LCN-2-positive signal normalised to the total number of cells confirmed a significant increase in LCN-2 protein expression in ccRCC samples (Fig. 2a, right), which was associated with tumour grade (Fig. 2b) and pT stage (Fig. 2c). Taking the hypothesis into account that the iron load might determine the biological function of LCN-2,^18,23,24^ we next aimed at identifying the iron status of tumour tissue as well as the LCN-2 iron load in ccRCC tissue. To determine LCN-2R expression, which is relevant for the uptake of LCN-2, we analysed LCN-2R mRNA (Fig. 2d) and protein (Fig. 2e) expression in whole-tissue homogenates from renal cancer samples compared with matched healthy adjacent control tissue. *LCN-2R* mRNA levels were significantly induced in ccRCC tissues (Fig. 2d). To compare LCN-2R protein expression in ccRCC samples with healthy adjacent tissue, CD326^+^ epithelial cells of healthy and tumour tissue were analysed by flow cytometry (Fig. 2e). CD326^+^ tumour cells expressed significantly more LCN-2R. To determine the functional outcome of enhanced *LCN-2R* expression in our cohort for patient prognosis, the cancer genome atlas data set of 533 ccRCC samples was analysed with regard to survival (Fig. 2f). Corroborating our own observations, higher *LCN-2R* expression correlated with lower overall survival probability analysed by the R2: Genomics Analysis and Visualization Platform applying the ‘Tumor Kidney Renal Clear Cell Carcinoma - TCGA – 533’ data set.

### LCN-2 is iron loaded in ccRCC compared with adjacent healthy renal tissue

**Fig. 3:**
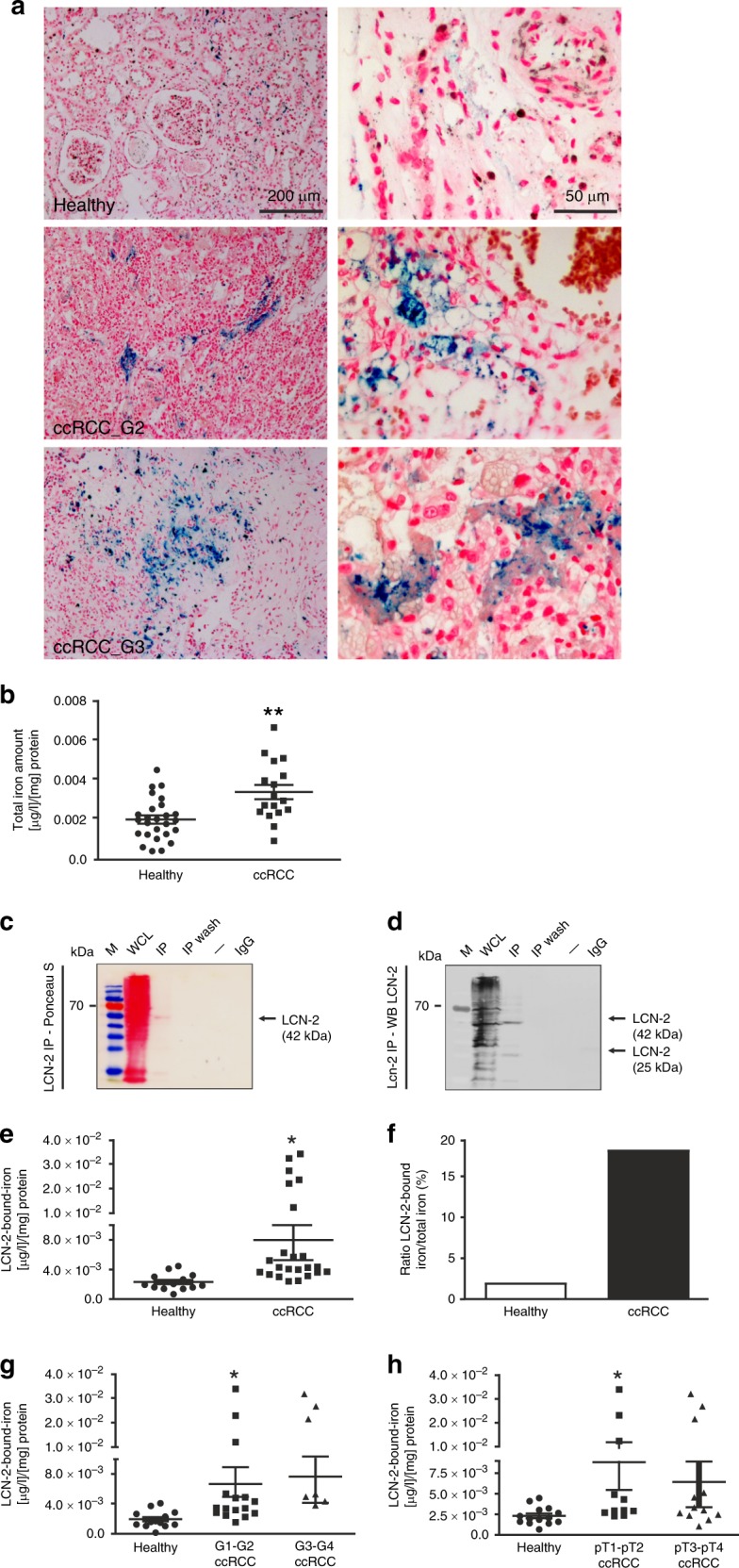
LCN-2 IP of whole-tissue homogenate reveals higher LCN-2-bound iron in total ccRCC tissue. **a** Perl’s stain of ccRCC tumour tissue and healthy adjacent tissue (representative pictures of two patients from *n* ≥ 11). **b** Quantification of total iron amount by atomic absorption spectrometry in total tissue homogenates from renal tumour and adjacent healthy tissues. **c**, **d** Controls for IP (IP) are whole-cell lysate (WCL), which is subjected to IP, the last washing step of the IP (IP wash) and beads treated with isotype IgG (IgG) instead of specific LCN-2 antibody. M (marker) indicates the size marker in kDa and – an empty lane. **c** Representative Ponceau-S stain of patient samples undergoing LCN-2 IP. The band of the 42-kDa LCN-2 dimers is marked by an arrow. **d** Immunological detection of LCN-2 by western blot. LCN-2 monomer (25 kDa) and LCN-2 dimer (42 kDa) are indicated by arrows. **e** Subsequent AAS of IP samples comparing LCN-2-bound iron of healthy and ccRCC tissue (*n* ≥ 14). **f** Fraction of LCN-2-bound iron in the total iron pool in whole-tissue homogenates. **g** LCN-2-bound iron of healthy tissue, G1–G2 and G3–G4 tumour grade, determined by AAS (*n* ≥ 14). **h** Iron amount bound by LCN-2 in healthy, pT1–pT2 and pT3–pT4 samples (*n* ≥ 14). Graphs are displayed as means ± SEM with *p < 0.05.

First, we stained iron deposits in ccRCC tissues by using Perl’s staining (Fig. 3a). We found significantly more iron-rich areas in tumour tissue than in adjacent healthy tissue. We quantified the iron amount of whole-tissue homogenates by applying atomic absorption spectrometry (AAS) (Fig. 3b) and confirmed higher iron amounts in tumour tissue than in adjacent healthy tissue. Since we previously described LCN-2 as an iron transporter in the tumour context,^8,9,19^ we next aimed at identifying the LCN-2 iron load in both healthy and tumour tissue. Therefore, we first established immunoprecipitation (IP) of LCN-2 by using primary human tissue. After immunoprecipitation and gel-electrophoretic separation of samples, Ponceau-S staining was used to control specificity, showing only defined bands in the IP sample (Fig. 3c). Western blot analysis confirmed specificity of the IP, since LCN-2 was exclusively detected in the IP sample at its predicted sizes of 25-kDa monomer and 42-kDa dimer (Fig. 3d). In order to quantify LCN-2-bound iron, AAS was performed by using IP samples. We detected significantly enhanced LCN-2-bound iron in tumour tissue compared with healthy controls (Fig. 3e). Taken together, LCN-2-bound iron accounts for ~2% in healthy tissue as compared with ~20% in tumour tissue (Fig. 3f). By comparing the LCN-2 iron load with tumour grade (Fig. 3g) and pT stage (Fig. 3h), we noticed an association of high LCN-2 iron amount to enhanced grade and stage.

### The iron load of LCN-2 determines its pro-tumour function

**Fig. 4:**
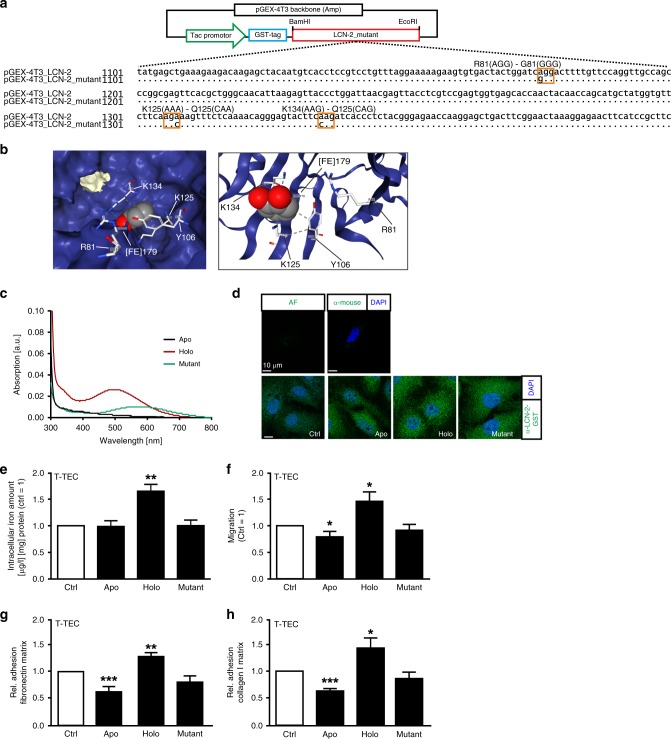
LCN-2 delivers iron to cells and promotes tumour growth. **a** Schematic overview of pGEX-4T3_LCN-2 template and its point mutations of amino acids involved in iron chelation to generate an LCN-2 triple mutant (R81→G, K125→Q, K134→Q). The LCN-2 sequence is flanked by *BamHI* and *EcoRI* restriction sites. N-terminal gluthathione S-transferase tag (GST) serves for purification. Point mutations are highlighted by boxes. **b** RCSB PDB Protein data bank was used to generate the model of LCN-2 iron binding by using the PDB code 3FW4. The model shows the complex of LCN-2 with ferric catechol. **c** UV–Vis absorption spectra of apo- (iron free, black), holo- (iron loaded, red) and iron-loaded triple mutant LCN-2 (mutant, green) show different absorption spectra [arbitrary units, a.u.]. Spectra were detected between 300 and 800 nm. **d** LCN-2 uptake was investigated by immunofluorescence. Autofluorescence (AF) and secondary antibody stain (α-mouse) served as controls for specificity. Except for AF image, nuclei were stained with DAPI (blue). Recombinant LCN-2 is detected via its GST tag (green). CAKI1 cells were stimulated with apo-, holo- and iron-loaded mutant LCN-2 in comparison with untreated (Ctrl) cells (*n* ≥ 3; five pictures each; 1 µg/ml LCN-2 for 24 h). **e** Intracellular iron amount of T-TEC cells was determined by AAS after stimulation with apo-, holo- or iron-loaded mutant LCN-2 (*n* ≥ 3). **f** T-TEC cells were stimulated with apo-, holo- or iron-loaded mutant LCN-2 and migration was recorded (*n* ≥ 3 with three technical replicates). **g**, **h** Matrix adhesion was probed after 24 h of stimulation with 1 µg/ml apo-, holo- or iron-loaded mutant LCN-2 to (**g**) fibronectin- or (**h**) collagen I-coated plates (*n* ≥ 3 with three technical replicates and five pictures each). Graphs are displayed as means ± SEM, with *p < 0.05, **p < 0.01, ***p < 0.001.

In order to test the assumption that LCN-2 transports iron to tumour cells, we generated a recombinant LCN-2 mutant protein (Fig. 4a). The positively charged amino acids arginine 81 (R81), lysine 125 (K125) and lysine 134 (K134) within the LCN-2 calyx confer binding of iron, as they form non-covalent cation–π interactions with electron-rich, aromatic structures of catecholate-type low-molecular-weight molecules, such as bacterial siderophores.^25^ These residues were mutated into non-charged ones (arginine to glycin, and lysin to glutamine). A scheme of the newly generated plasmid is depicted in Fig. 4a and the model of iron–catechol binding within the LCN-2 calyx applying the PDB code 3FW4 is depicted in Fig. 4b. The iron-binding ability of the recombinant mutant protein was monitored by UV–Vis spectrometry (Fig. 4c). Iron-free (apo-) LCN-2 served as a reference and showed no peak between 400 and 700 nm (black line). Upon iron loading, holo-LCN-2 displayed a maximum at 497 nm (red line), whereas iron loading of the mutant LCN-2 protein displayed a shift of the peak to 572 nm (green line), indicating substantially impaired iron binding. To test whether recombinant proteins are taken up by tumour cells, renal ccRCC CAKI1 cells were stimulated with apo-, holo- and mutant LCN-2. In order to exclusively detect LCN-2 taken up by tumour cells, we used recombinant proteins with a GST tag and immunofluorescence detection (Fig. 4d). We observed that iron-free (apo), iron-loaded (holo) as well as the mutant protein were internalised by tumour cells in comparison with unstimulated controls and uptake occurred to a similar extent. To clarify whether LCN-2 effectively delivered iron to tumour cells, we isolated primary human patient-derived tumour cells (T-TEC) and measured cell lysates of apo-, holo- and mutant LCN-2-stimulated T-TEC by AAS for their iron amount in comparison with unstimulated cells (Ctrl) (Fig. 4e). As expected, only holo-LCN-2 increased the intracellular iron amount. In contrast, stimulation with apo-LCN-2 or the mutant protein remained without effect regarding the intracellular iron amount.

Since we found elevated levels of iron-loaded LCN-2 in ccRCC patients, we aimed at identifying if LCN-2-dependent pro-tumour effects are mediated through its iron load. Migration of primary human T-TEC was significantly reduced upon stimulation with apo-LCN-2, while holo-LCN-2 significantly induced tumour cell migration. The mutant LCN-2 had no effect (Fig. 4f). Similarly, adhesion to fibronectin (Fig. 4g) or collagen I (Fig. 4h) matrices was significantly enhanced upon holo-LCN-2 stimulation, whereas apo-LCN-2 blocked adhesion.

**Fig. 5:**
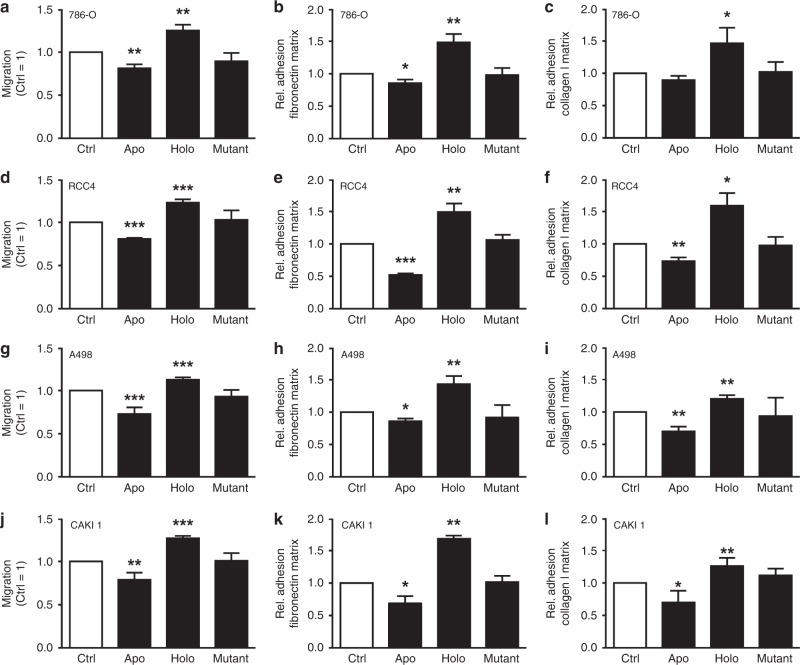
LCN-2 acts as a pro-tumorigenic agent in multiple cancer cell lines. **a**–**l** Cell lines were stimulated with 1 µg/ml apo-, holo- or iron-loaded mutant LCN-2. (Left panels) Migration was recorded from at least five independent experiments performed with three technical replicates each. Slope [1/h] of migration was normalised to unstimulated (Ctrl) cells. (Middle and right panels) For adhesion, cell lines were stimulated for 24 h with 1 µg/ml apo-, holo- or iron-loaded mutant LCN-2, and adhesion to coated matrices was probed in comparison with unstimulated cells (Ctrl). Adherent cell numbers were normalised to control treatments. Adhesion assays were performed at least in five independent experiments. Five pictures of each of the three technical replicates were acquired and analysed. **a**–**l** Migration and adhesion to fibronectin or collagen I matrix of LCN-2 stimulated of (**a**–**c)** 786-O, (**d**–**f)** RCC4, (**g**–**i)** A498 and (**j**–**l)** CAKI1 (*n* ≥3). Graphs are displayed as means ± SEM, with *p < 0.05, **p < 0.01, ***p < 0.001.

**Fig. 6:**
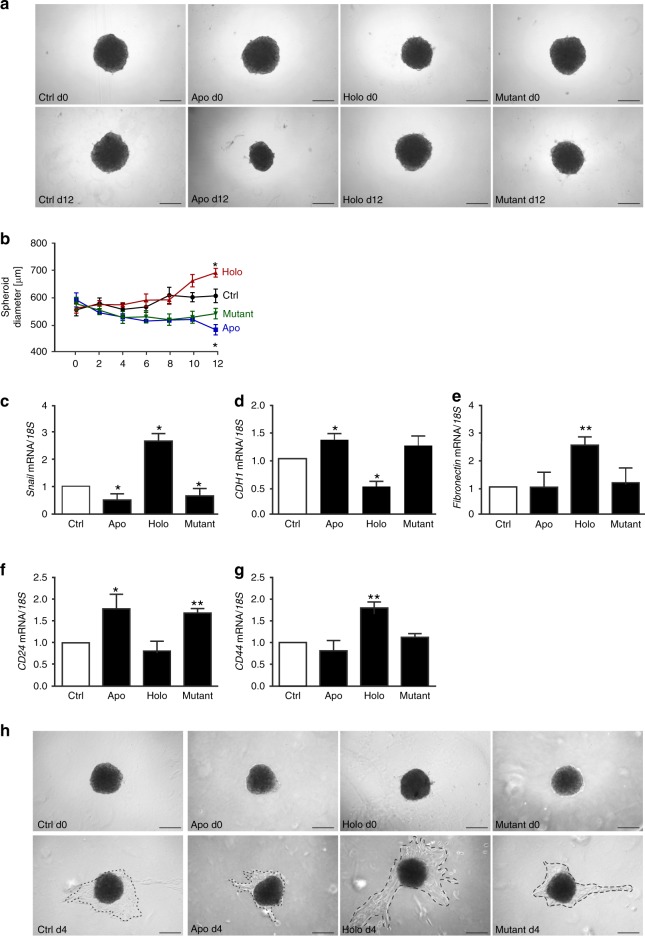
LCN-2 affects tumour growth and progression in a 3D spheroid model. CAKI 1 renal carcinoma cells were grown as 3D spheroids, and were either left untreated (Ctrl) or stimulated with apo-, holo- or mutant LCN-2 (1 µg/ml) over a period of 12 days. *n* = 4 independent experiments with five technical replicates each. **a** Representative pictures of days 0 and 12 are given for each condition. Scale bar: 250 µm. **b** Quantitative analysis of spheroid growth was assessed by using ImageJ software and is displayed as spheroid diameter (µm). **c**–**g** Spheroids were picked at day 12 and dissociated by using accutase for further RNA processing. qRT-PCR analysis for (**c**) *Snail*, (**d**) *CDH1*, (**e**) *fibronectin*, (**f**) *CD24* and (**g**) *CD44*, normalised to the housekeeping gene *18* *S*. **h** CAKI 1 spheroids were treated with apo-, holo-, or mutant LCN-2 (1 µg/ml) every second day, or remained unstimulated (Ctrl) for 12 days and were embedded into a collagen I matrix for additional 4 days. Representative pictures are shown from day 0 directly after embedding the spheroids into the collagen I matrix and day 4. The distance of spheroid cell invasion from the spheroid body into the matrix is depicted by the punctuated line. The assay was performed in four independent experiments with three technical replicates. Scale bar: 250 µm. Graphs are displayed as means ± SEM, with *p < 0.05, **p < 0.01, ***p < 0.001.

In order to test if the observed effects could be extended to other cell lines, we tested the ccRCC cell lines 786-O (Fig. 5a–c), RCC4 (Fig. 5d–f), A498 (Fig. 5g–i) as well as CAKI 1 (Fig. 5j–l). In all cell lines, we observed that iron-free LCN-2 reduced migration and matrix adhesion. In contrast, stimulation with iron-loaded LCN-2 enhanced migration and adhesion. Cellular proliferation remained unaltered upon stimulation with apo-, holo- or mutant LCN-2 protein (Supplementary. Fig. S1). Next, we analysed the effects of LCN-2 in a physiologically relevant 3D spheroid model (Fig. 6). CAKI 1 tumour spheroids were stimulated with 1 µg/ml apo-, holo- or the non-iron-binding LCN-2 mutant every second day over a period of 12 days. Growth characteristics were determined by analysing the spheroid diameter. Representative pictures are given in Fig. 6a. Software-based analysis of the spheroid diameter (Fig. 6b) showed a significantly reduced tumour growth upon apo-LCN-2 stimulation, whereas holo-LCN-2 enhanced spheroid size. Stimulation with the mutant LCN-2 showed similar effects as apo-LCN-2. Since we previously observed a strong effect of holo-LCN-2 on tumour cell migration (Fig. 5), we then performed qRT-PCR analysis of migration-associated genes, such as the transcription factor *Snail* (Fig. 6c), the epithelial marker *CDH1* (Fig. 6d), the mesenchymal marker *fibronectin* (Fig. 6e) as well as the stemness markers *CD24* (Fig. 6f) and *CD44* (Fig. 6g). Holo-LCN-2 generally provoked an EMT (epithelial-to-mesenchymal transition) phenotype with enhanced *Snail*, *fibronectin* and *CD44* expression, while expression of *CDH1* and *CD24* was reduced. In contrast, apo-LCN-2 showed the opposite effect. The non-iron-binding mutant LCN-2 followed the same pattern as apo-LCN-2. In order to test if these observations translate into functional consequences, we performed a spheroid invasion assay (Fig. 6h). We stimulated CAKI 1 spheroids over a period of 12 days every second day with apo-, holo- or the mutant LCN-2 (1 µg/ml). After this stimulation period, we transferred spheroids into a collagen I matrix and allowed them to spread for additional 4 days. We found that the holo-LCN-2 stimulation significantly promoted the dissemination and invasion of cells from the spheroid into the collagen matrix, whereas apo- and mutant LCN-2-stimulated spheroids remained unaltered compared with control spheroids.

## Discussion

We present evidence that LCN-2, specifically iron-loaded LCN-2, is significantly elevated in tumour tissue isolated from patients with renal cancer as compared with adjacent healthy tissue. As LCN-2 was found in its iron-bound form in primary human renal cancer samples, and its presence being associated with tumour grade and stage, we further investigated the role of the LCN-2 iron load in determining its pro-tumour function.

While the importance of LCN-2 during the development of acute and chronic renal pathologies is widely accepted, its role in renal tumours still remains largely elusive. Previous studies identified increased urinary and serum LCN-2 in RCC patients without an association to disease type, tumour stage or grade.^14,15^ However, tissue LCN-2 protein positively correlated with histological grades of ccRCC and pRCC.^26^ Due to the low sample number for high-grade tumours, we could not draw any significant conclusion if LCN-2 protein might be associated with tumour grade and/or stage, which remains to be addressed in further studies. Interestingly, *LCN-2* mRNA was not significantly elevated in ccRCC samples and did not show any association to tumour grade or stage. By analysing the TCGA KIRC data set, we found reduced *LCN-2* expression in ccRCC patients. However, we observed two populations of patients in our cohort: one with a high *LCN-2* mRNA expression and one with low *LCN-2* mRNA expression. The apparent clustering still remains to be investigated in detail by using higher sample numbers for high-grade tumours. Since previous studies in renal cancer pointed to a close association of high LCN-2 expression and reduced progression-free survival in pRCC,^27^ ccRCC^28^ and sunitinib-treated RCC patients,^29^ it might be very interesting to follow-up the association of these high LCN-2-expressing tumours with overall or progression-free survival. These observations are in accordance with the role of LCN-2 as a pro-tumour factor in a variety of other urogenital neoplasms, including prostate,^30^ endometrial,^31^ ovarian^32^ and breast cancer.^11^ However, the underlying mechanisms of its pro-tumour activity are still poorly understood.

We recently found that the unique iron-transporting capacity of LCN-2 is of crucial importance for its pro-tumour activity. Blocking LCN-2 iron release from TAM significantly reduced mammary tumour growth.^19^ Due to their high proliferative rate, cancer cells require high amounts of iron in order to sustain their enhanced metabolic turnover.^33^ Hence, tumour cells show enhanced iron retention, which results in the development of more aggressive tumours.^34^ In this context, a large number of studies revealed the role of iron-trafficking proteins in the context of cancer, associating the overexpression of transferrin receptor (TfR),^35^ ferritin^36^ or the iron-regulatory protein-2^37^ with a higher tumour grade and increased chemoresistance. Whether LCN-2 also has to be included as an iron-trafficking protein in the context of cancer, still needs detailed analysis of its mechanism of action and its iron-transporting as well as iron-donating function. Interfering with the iron availability reduced growth and progression in both human and mouse mammary carcinomas.^38^ Interestingly, repeated systemic iron administration enhanced deposition of iron in the kidney, which induced oxidative stress in renal tubular epithelial cells, in turn, fostering the development of RCC.^39^ Since iron availability is tightly interlinked with the physiology of the hypoxic response, it is crucial to control the expression of iron-regulated genes, one of which might be LCN-2. Importantly, the transcriptional activity of hypoxia-inducible factor (HIF)-α is of central importance to the development of ccRCC. HIF-α stability is regulated by the von Hippel Lindau (VHL) protein. Interestingly, the VHL gene is mutated or hypermethylated in about 90% of all ccRCC patients, with its loss of function leading to the accumulation of HIF-α.^1^ In turn, HIF-α is essential to regulate the expression of a variety of iron-regulated genes, including TfR, ferritin and ferroportin.^40^ Thus, the HIF-α signalling pathway might be essential to control cellular iron trafficking and mobilisation, which is important to promote tumour growth. Moreover, it was previously described that LCN-2 is able to induce tumour neovascularisation via the HIF-1α-VEGF axis,^12^ which adds to the therapeutic potential of LCN-2 in renal tumours.

However, we speculate that a more potent treatment strategy would imply inhibition of the tumour iron supply in order to deprive tumour cells of this essential nutrient. In this context, we recently described TAM as a pivotal source of iron in the tumour microenvironment by adopting an iron-release phenotype during tumour growth.^19^ Interfering with the iron-release phenotype by using iron chelators^41^ significantly reduced the pro-tumour effect of TAM. Renal TAM also releasing LCN-2 is still not clearly defined and is currently under investigation. However, since LCN-2 is a secreted protein, the possibility exists that iron-loaded LCN-2 is rapidly captured by tumour cells due to the enhanced expression of SLC22A17, the LCN-2 receptor. Indeed, we observed enhanced expression of *SLC22A17* in ccRCC patients in our cohort, thus pointing towards the possibility that the LCN-2 receptor might play an important role. Given the observation that the LCN-2 iron-loaded form was significantly elevated in ccRCC patients, we proposed that the iron load defines the pro-tumour characteristics of LCN-2 in renal cancer. We tested this hypothesis by generating a mutant LCN-2 protein, deficient of its iron-binding capacity. Iron-loaded (holo-) LCN-2 enhanced migration and matrix adhesion of a variety of ccRCC cell lines as well as primary patient-derived tumour cells, while the iron-free (apo-) protein significantly reduced migration and adhesion of all tumour cells investigated. However, it was surprising that the mutant LCN-2 form, which is unable to bind iron, differed significantly from the apo form of LCN-2. It was previously described that apo-LCN-2 binds iron within the cell and exports it to the outside, whereby cells are deprived of iron, and as a result, become apoptotic.^18^ In our set-up, we ruled out this possibility due to the fact that the intracellular iron amount of cells treated with either apo- or the mutant LCN-2 did not differ. We assume that intracellular iron is captured by the apo form and no longer available for cell growth. In the case of the mutant form, no iron binding is possible, and thus, no antitumour effect is observed. These considerations still need experimental proof. Interestingly, cellular proliferation remained without an effect for both holo- and apo-LCN-2. These observations suggest that the pro-tumour effects of LCN-2 are more potent during later phases of tumour development and/or during the metastatic process. By employing physiologically more relevant 3D tumour models, we confirmed these observations.

Our study underscores the significance of the iron load in defining the pro-tumour characteristics of LCN-2. However, detailed mechanistic insights are still lacking and need to be defined before transferring these observations into clinically relevant tumour models.

## Supplementary information

Supplemental Figure legend

Supplemental Figure S1

## Data Availability

The data sets used and/or analysed during this study are available from the corresponding author on reasonable request. Publicly available data sets were analysed by using R2: Genomics Analysis and Visualization Platform (Academic Medical Center—Department of Oncogenomics, Amsterdam, 2006, http://r2.amc.nl), or downloaded from the cBioPortal for Cancer Genomics (https://www.cbioportal.org) and analysed by using Prism software.
