# Supplementary material for: The iron load of lipocalin-2 (LCN-2) defines its pro-tumour function in clear-cell renal cell carcinoma
Source: Br J Cancer. 2019 Nov 27;122(3):421–33. doi: 10.1038/s41416-019-0655-7 (PMC7000824; doi:10.1038/s41416-019-0655-7)
Supplement: Supplementary file 1 — Supplemental Figure legend [file 41416_2019_655_MOESM1_ESM.docx]

**Supplemental Figure S1: Effects of LCN-2 on cellular proliferation**

Proliferation of (A) CAKI 1 cells and (B) T-TEC upon stimulation with either apo-, holo-, or mutant LCN-2 protein (1 µg/ml) was determined on an xCELLigence instrument and is given relative to untreated control (Ctrl) cells (n=4). Data are means ± SEM from independent experiments with 3 technical replicates.
